# Does Smoking Protect against Being Hospitalized for COVID-19?

**DOI:** 10.3390/ijerph17249559

**Published:** 2020-12-21

**Authors:** Ivan Berlin, Daniel Thomas

**Affiliations:** 1 Department of Pharmacology, Hôpital Pitié-Salpêtrière-Assistance Publique-Hôpitaux de Paris, 75013 Paris, France; 2 Centre Universitaire de Médecine Générale et Santé Publique, UNISANTE, 1010 Lausanne, Switzerland; 3 Institut de Cardiologie, Hôpital Pitié-Salpêtrière-Assistance Publique-Hôpitaux de Paris, 75013 Paris, France; thomas.daniel@neuf.fr

Gonzalez-Rubio et al. [1] compared the prevalence of current smoking among those hospitalized for COVID-19 to smoking prevalence in the general populations of China, USA, and Italy, and reported a substantially reduced prevalence among hospitalized individuals compared to the countries’ general prevalence of smoking (expected prevalence). They concluded that their results “confirmed the protective effect of current smoking on the likelihood of hospitalization” and provided a series of potential mechanisms by which tobacco-smoking could be protective against COVID-19.

This paper raises serious concerns. It is well-documented that the data collection on smoking status among hospitalized individuals and in particular from electronic reports is poor [2]. None of the included papers reported on biochemical verification of smoking status. Some potential biases are also mentioned by the authors, such as smokers could “hide” their smoking status when hospitalized; some may have stopped when they became ill and were counted as nonsmokers; the definition of “current smoker” is not always provided. They also acknowledged that case-control studies, such as those included in this systematic review, erroneously concluded about a “protective” effect of smoking in Alzheimer’s disease.

More importantly, individuals hospitalized for COVID-19 cannot be compared with confidence to a general population of a country. None of the population characteristics can be similar, the comparison remains historical, and it can even not be considered as a case-control design. It is not surprising that the prevalence rate of smoking is substantially lower among individuals hospitalized for COVID-19 in each country than in their general populations and shows a relatively low measure of dispersion. These may suggest a systematic error and highly compromise the conclusions.

A recent report shows that substance-use disorders largely increase the risk of COVID-19 and in particular, tobacco-use disorder increases the risk by 8-fold (adjusted odds ratio = 8.222; 95% confidence interval: 7.925–8.530) [3]. Moreover, as could be expected, according to the most recent meta-analysis of 47 studies, patients with a smoking history have an increased risk of severe COVID-19 (risk ratio (RR): 1.31; 95% CI: 1.12–1.54), in-hospital mortality (RR: 1.26; 95% CI: 1.20–1.32), disease progression (RR: 2.18; 95% CI: 1.06–4.49), and need for mechanical ventilation (RR: 1.20; 95% CI: 1.01–1.42) [4].

As Griffith et al. [5] point out, collider bias may undermine our understanding of COVID-19 risk, and the supposedly protective effect of smoking is one example [5].

Speculation about biological mechanisms is adequate only if a cause-to-effect relationship between smoking and hospitalization for COVID-19 is demonstrated in prospective studies. A protective effect should be evoked with extreme caution [6] because a biased message may induce smokers to continue to smoke or to relapse to smoking to protect themselves from COVID-19.
